# Helminths and HIV infection: epidemiological observations on immunological hypotheses

**DOI:** 10.1111/j.1365-3024.2006.00904.x

**Published:** 2006-11

**Authors:** M BROWN, P A MAWA, P KALEEBU, A M ELLIOTT

**Affiliations:** 1 London School of Hygiene & Tropical Medicine London, UK; 2 Medical Research Council/Uganda Virus Research Institute, Uganda Research Unit on AIDS Entebbe, Uganda

**Keywords:** helminth, HIV, immuno-regulation

## Abstract

Parasitic helminths have co-evolved with the mammalian immune system. Current hypotheses suggest that immunological stimulation in the presence of helminths is balanced by immuno-regulation and by the broad spectrum of mechanisms possessed by helminths for countering the host immune response. The degree to which this balance is perfected, and the mechanisms by which this is achieved, vary between helminth species; we suggest that this is reflected not only in the degree of pathology induced by helminths but also in a variety of relationships with HIV infection and HIV disease. Available epidemiological data regarding interactions between helminths and HIV are largely observational; results are variable and generally inconclusive. Well designed, controlled intervention studies are required to provide definitive information on the species-specific nature of these interactions and on the advantages, disadvantages and optimal timing of de-worming in relation to HIV infection.

## INTRODUCTION

Helminth and Human Immunodeficiency Virus (HIV) infection have major effects on the host immune response and co-infection is widespread (1). They are, however, quite different in respects that are important to their interactions.

First, helminth infections are caused by diverse species from three phyla: generalizations regarding the mechanism and nature of their effects may not be appropriate. HIV is essentially a single entity.

Second, helminth infection may be, in a sense, normal to the mammalian immune system. The association between mammals and helminths is ancient: some species probably co-evolved with primates and humans; others, co-evolved with other mammals, may have crossed and adapted to humans following exposure through domestication of animals. In some host–helminth relationships adaptation appears almost perfect. Most people with helminths are unaware of their infection: the persistence of widespread infection, despite disease caused by some high intensity infections, is compatible even with a selective advantage of low intensity infection to the host. By contrast, HIV infection is not normal; it has attained a significant prevalence in humans only in the last 25 years and is universally fatal.

Third, helminths modulate the immune system, but HIV destroys it. Immune modulation by helminths may have both beneficial and detrimental effects in relation to human disease. There is evidence for benefit of helminth infection in relation to atopic disease (2,3) and to the inflammatory pathology of autoimmune disease (4–6). Studies of co-infection with helminths and malaria have produced conflicting results and interpretation of the findings varies, but it is possible that helminths may be associated with some protection against cerebral malaria, although control of replication of the parasite may be impaired (7,8). On the other hand, for bacterial and viral infections, impaired control of replication and elimination of infection may generally lead to a detrimental outcome (9–12). That HIV infection is detrimental to the immune response to many pathogens is quite clear and poor regulation of the immune system in advanced HIV infection is illustrated by an increased incidence of hypersensitive drug reactions (13,14).

## IMMUNOLOGICAL HYPOTHESES

During the 1990s it was proposed that helminths might impair the immune response to HIV, leading to greater susceptibility to HIV acquisition and more rapid HIV progression (15). This hypothesis was based on the concept that a T helper (Th)2 bias, induced by helminths, was a form of immune ‘dysregulation’ that might be detrimental in several ways. These included promotion of a Th2 bias with suppression of protective, Th1 responses to HIV; and expansion of a population of Th2 lymphocytes that were more susceptible to HIV infection (16); cells might also be more susceptible to HIV infection due to increased HIV coreceptor expression in response to immune activation (17,18). However, the apparent health of many helminth-infected people reflects the fact that pathways activated by helminth infection tend to be balanced by regulatory mechanisms, with recent interest focused particularly on the role of regulatory CD4^+^CD25^+^ T cells, interleukin (IL)-10 and transforming growth factor (TGF)-β (2,19). The degree to which this balance is perfected, allowing long-term parasite survival with minimal damage to the host, varies and may be enhanced in individuals born to helminth-infected mothers compared to those first exposed later in life (20,21). Thus the sequelae of infection range from an apparently healthy host despite heavy intensity infection (as with the filarial infection, *Mansonella perstans*, and the intestinal parasite, *Enterobius vermicularis*) to the severe disease manifestations of advanced schistosomiasis; this spectrum attests to the complex interactions between effective and regulated immune responses and destructive immunopathology, which continue to mould the ecological relationship between humans and helminths. With the recent introduction of HIV into these long-established systems, pathways involved in both pro-inflammatory and regulatory responses may influence the outcome of HIV or of helminth exposure and infection.

Helminth-induced immuno-regulatory mechanisms that suppress protective responses to HIV could be detrimental. Although the function, phenotype and antigen-specificity of CD4^+^CD25^+^ regulatory T cells is not yet well defined, they have been implicated in the modulation of immune responses to bystander antigens (22) and could suppress HIV-specific CD4^+ - and^ CD8^+^ -derived cytokine production and lymphocyte proliferation, suggesting that they may play a role in suppressing antiviral immune responses (23–28). On the other hand, regulatory activity could have benefits: replication of proviral DNA depends on activation of host cell transcription factors (29), so helminth-induced regulatory activity that suppresses such transcription could be beneficial, particularly in relation to HIV progression (30).

Conversely, a reduction in numbers or function of regulatory T cells has been observed in advanced HIV disease (31,32) and high foxp3 expression (indicative of regulatory T cells) has been found to correlate inversely with markers of immune activation, suggesting that loss of these cells may reduce suppression of immune activation. Regulatory T cells express the HIV coreceptor CCR5 and are readily infected by HIV *in vitro* (31): perhaps they are preferentially eliminated by direct HIV infection leading to uncontrolled immune activation and dysfunction. This might have important implications for the host–parasite interaction: could preferential depletion of regulatory cells, for example, create an environment inimical to parasite survival and reproduction?

Thus immunological data now suggest a range of scenarios in which helminths and HIV may each either promote or oppose acquisition or progression of the other condition.

## EPIDEMIOLOGICAL OBSERVATIONS

Initial hypotheses regarding helminths and HIV were developed largely in the absence of epidemiological data to indicate whether substantial effects occurred, and amid controversy as to whether HIV progression was actually more rapid in regions where helminth prevalence was high (33). Several studies have now been conducted. To date, only one randomised trial of the effects of treatment (34) has yet been reported. It must be anticipated that effects are bi-directional: the effects of helminths on the immune system may influence the outcome of exposure to and infection with HIV, and the effects of HIV on the immune system may influence the outcome of exposure to and infection with helminths. Further, particularly in the case of cross-sectional and observational studies, results must be interpreted in the light of potential confounding, and of the progressive nature of HIV disease.

### Effects of helminths on HIV

#### Effects of helminths on susceptibility to HIV infection

Studies that have examined co-prevalence of HIV and helminth infections are summarized in Table 1. The findings are pertinent to whether helminths increase susceptibility to HIV, or HIV increases susceptibility to helminths. In either case, a higher prevalence of HIV might occur in individuals with helminths.

**Table 1:** Associations between helminth infections and HIV status

	Helminth prevalence observed by HIV status				
				
Worm species	HIV-positive	HIV-negative	*P*	Country (ref)	Comment
*S. haematobium*	*n* = 134	*n* = 345		Zimbabwe (35)	Study in women only
in urine	45%	37%	NS		
in Papanicolaou smear	10%	5%	0·04		
in any genital specimen	23%	13%	0·008		
	*n* = 83	*n* = 166		Kenya (36)	Case control study in pregnant women ^a^predominantly hookworm
*Wuchereria bancrofti*	29/80 (36%)	22/155 (14%)	< 0·001		
Schistosomiasis	5/63 (8%)	12/122 (10%)	NS		
Intestinal helminths^a^	24/57 (42%)	43/89 (48%)	NS		
	*n* = 100	*n* = 85^b^		Brazil (37)	^b^Healthy volunteers; presumed HIV-negative
*Strongyloides*	12%	7%	NS		
hookworm	4%	7%	NS		
	*n* = 407	*n* = 1138		Zimbabwe (38)	
*S. haematobium*	27%	28%	NS		
*S. mansoni*	10%	7%	NS		
*S. haematobium* & *mansoni*	9%	8%	NS		
*Strongyloides*^c^	*n* = 78	*n* = 78		Thailand (39)	^c^When stratified for presence of diarrhoea, association with *Strongyloides* seen only in people without diarrhoea
*Opisthorchis*	18%	8%	< 0·05		
	19%	19%	NS		
	*n* = 78	*n* = 26		Ethiopia (40)	All participants had diarrhoea
*Ascaris*	31%	23%	NS		
*Strongyloides*	5%	4%	NS		
*Trichuris*	6%	8%	NS		
*S. mansoni*	3%	4%	NS		
hookworm	3%	4%	NS		
	*n* = 211	*n* = 213		Brazil (41)	
*Strongyloides*	10%	6%	NS		
*Ascaris*	2%	5%	NS		
	*n* = 365	*n* = 5243		Brazil (42)	
*Ascaris*	12%	9%	NS		
*Trichuris*	5%	6%	NS		
hookworm	4%	4%	NS		
*S. mansoni*	2%	2%	NS		
*Strongyloides*	6%	1%	< 0·001		
	*n* = 52	*n* = 1187		Ethiopia (43)	
*S. mansoni*	25%	32%	NS		
	*n* = 52	*n* = 48		Honduras (44)	
*Trichuris*	21%	40%	0·05		
hookworm	17%	8%	NS		
*Strongyloides*	8%	0%	NS		
*Ascaris*	2%	21%	0·003		
	*n* = 93	*n* = 2093		Uganda (45)	NS after adjusting for geographical location and duration of residence.
*Onchocerca*	78%	88%	0·015		
	*n* = 112	*n* = 239		Tanzania (46)	
*ascaris*	4%	10%	0·04		
*Strongyloides*	10%	1%	< 0·001		
hookworm	12%	13%	NS		
*S. haematobium*	*n* = 49	*n* = 846		Congo (47)	
by serology	16%	26%	NS		
by egg count in urine	10%	18%	NS		

*S. mansoni: Schistosoma mansoni; S. haematobium: Schistosoma haematobium*. NS: no statistically significant effect.

Until recently, published studies consistently indicated a lack of epidemiological association between schistosomiasis and HIV. This was particularly surprising for *Schistosoma haematobium*, where the presence of female genital lesions and of blood and leucocytosis in seminal fluid (48) were expected to promote transmission, regardless of possible immunological effects (49). However, a recent study among rural Zimbabwean women showed a significant association between HIV and the presence of *S. haematobium* ova in genital samples, supporting the hypothesis of a specific effect on susceptibility to HIV when genital lesions are present (35).

Among nematodes, apart from one study in pregnant women indicating an association between HIV and *Wuchereria bancrofti* (36), the only evidence of a positive association is for *Strongyloides stercoralis* (39,42,46). *Strongyloides* is unusual among helminths in that it is able to complete its life-cycle and replicate in an individual human host, and this may be facilitated by immunosuppression, as discussed below. Given the lack of observed associations between HIV and other intestinal nematodes, a permissive effect of HIV on *Strongyloides* may be the most likely direction of effect.

Other studies suggest a negative association between HIV and nematode infection (44,46), which could be consistent either with a *protective* effect of nematodes against HIV infection or with the generation, by advancing HIV infection, of an environment that is inimical to establishment of nematode infection, nematode development, or the production of eggs (or microfilariae). In other words, HIV might ‘protect’ against the establishment or survival of a mature nematode infection, or might lead to under-diagnosis due to reduced fecundity. This possibility has been examined in relation to schistosomiasis, as discussed below, but has not yet been examined in relation to nematodes. Alternatively, the explanation may lie in unexplored confounding factors such as age and poverty.

An increase in susceptibility to HIV infection caused by helminths could be obscured in co-prevalence studies if helminths were also associated with increased HIV progression rates and hence mortality, balancing or outweighing increased incidence through loss of co-infected individuals. In this case, increased HIV prevalence in helminth-infected individuals would be more prominent early in an HIV epidemic; but even studies conducted early in the HIV epidemic for their region fail to show a positive association. In a mature epidemic, a positive association might be seen in young age groups, the effect disappearing, or becoming negative with greater age (i.e. longer duration of HIV infection): this possibility has not been examined in reported studies.

Thus co-prevalence studies to date fail to support the hypothesis that helminths generally promote acquisition of HIV infection in adults. This may be, in part, because studies were not designed specifically to address this hypothesis. Prospective studies of the incidence of sexually acquired HIV infection have not generally investigated associations with helminths, and this issue has yet to be studied in animal models *in vivo* (12). An exception is the recent study on female genital schistosomiasis: after one year of follow-up, seven of 224 women who were HIV-negative at baseline had seroconverted; all of these had *S. haematobium-*related findings at baseline (five with genital lesions, two with ova in urine) compared to 65% of those who did not seroconvert (*P =* 0·098); this result, although inconclusive, again supports the hypothesis of a specific effect on susceptibility when genital lesions are present (35). One retrospective case-control study has examined the effect of maternal helminths on vertical HIV transmission. In this study 13 of 44 HIV-exposed infants were found to be HIV infected at 12–24 months of age, and a positive association was observed between vertical transmission and maternal lymphatic filariasis (*Wuchereria bancrofti*) (36). If confirmed in further studies and for other helminths, this effect would be of considerable public health importance and might be amenable to intervention by de-worming during pregnancy, which is now advocated (for considerations such as potential effects on maternal anaemia, rather than HIV infection) (50).

#### Effects of helminths on HIV disease progression

Cross–sectional studies of associations between helminth infection and severity of HIV disease, measured by CD4^+^ T cell count and HIV load, suffer from issues of interpretation that are similar to those for co-prevalence studies. Thus, the hypothesized adverse effect of helminths on HIV progression might predict lower CD4 counts and higher viral loads in co-infected individuals; but higher mortality in co-infected individuals could obscure or invert such an effect, while suppression of helminth development or egg production in individuals with advanced HIV disease could lead to under-diagnosis and a spurious impression of a protective effect of helminths. Confounding with behavioural and socio-economic factors could again provide misleading results.

Most studies so far have presented results for any helminth compared to no helminth infection. Overall, CD4^+^ T cell counts in co-infected individuals have been higher, or similar, to those in participants with HIV alone and viral load has been similar, at least after adjusting for measured confounding factors (Table 2). Thus, at a simplistic level, results do not support the hypothesis of an adverse effect of co-infection on HIV disease. Individual studies have addressed aspects of these associations in more detail. A potentially important consideration is the effect of intensity of helminth infection. Wolday and colleagues (54) measured intensity by combining egg counts from the helminth species encountered (predominantly *Trichuris* and *Ascaris*) and found that individuals in the lowest 33% for intensity had lower viral load than those with medium or highest intensity infection (lower, too, than helminth-free participants, although the statistical significance of this difference was not presented). On the other hand, in Uganda, we found no correlation between intensity of *S. mansoni* infection and viral load (55); and a study in Zambia (predominantly *Ascaris* and hookworm) showed no statistically significant difference in viral load between moderate-to-high and low intensity infections (51). Another potentially important consideration is the effect of helminth species. Our results in Uganda suggested a possible distinction between species such as hookworm and *Mansonella*, where CD4^+^ T cell counts might be higher (and, in the case of *Mansonella*, viral load lower) and *Schistosoma* and *Strongyloides*, where CD4^+^ T cell counts might be similar (or, for *Strongyloides*, lower) and viral load higher (52). These observations require confirmation, as effects were, at best, marginally statistically significant, but serve to highlight the potential importance of effects of intensity, and of species-specific differences. Our study, conducted within an existing cohort, allowed a retrospective assessment of the effects of untreated helminths (compared to no helminths) on HIV progression measured by CD4^+^ T cell decline. No statistically significant effects were observed.

**Table 2:** Associations between helminth infections and CD4^+^ T cell count and viral load in HIV-positive subjects

Total number of HIV positive participants	Number infected and helminth species	Effect of co-infection with helminths on CD4^+^ T cell count	Effect of co-infection with helminths on viral load	Country (ref)	Comments
111	54 All species (predominant species: *Ascaris* and hookworm)	Higher (*P =* 0·02)	NS	Zambia (51)	
185	143 Schistosomiasis	NS	Not done	Zimbabwe (38)	Compared subjects with and without schistosomiasis
539	290 All species	NS	Higher (*P =* 0·03)^a^	Uganda (52)	^a^(NS afteradjustment for potential confounding factors)
	Hookworm	Higher (*P =* 0·007)	NS		
	*S. mansoni*	NS	NS		
	*Strongyloides*	NS	Higher (*P* = 0·02)^a^		
	*Mansonella*	Higher (*P* = 0·04)^a^	NS		
108	39 All species (predominant species: *S. mansoni* and hookworm)	Higher (*P =* 0·005)	NS	Uganda (53)	
56	31 All species (predominant species: *Trichuris* and *Ascaris*)	NS	NS	Ethiopia (54)	
365	161 All species^b^ (predominant species: *Ascaris*,*Strongyloides*,*Trichuris* and hookworm)	NS	Lower (*P* = 0·02)	Brazil (42)	^b^‘All species’ includes protozoa; information for all helminths without protozoa not given

*S. mansoni: Schistosoma mansoni; S. haematobium: Schistosoma haematobium.* NS: no statistically significant effect.

Prospective studies of the effects of treatment of helminths are summarized in Table 3. These require consideration of the progressive nature of HIV disease. So far the presumption that helminths are detrimental has generally led to the view that helminth infections, once identified, should be treated. Lawn and colleagues found that viral load increased progressively over 15 months after successful treatment of schistosomiasis with praziquantel, and that the increase was not related to the degree of decline in egg count or serum circulating cathodic antigen (CCA), suggesting, perhaps, a lack of association with the treatment. However, lack of a comparison group made it impossible to distinguish effects of HIV progression and praziquantel treatment (56). Kallestrup and colleagues addressed this issue by randomising participants with schistosomiasis and HIV infection to immediate vs. delayed praziquantel treatment. At 3 months they found no change in viral load in the treated group, but an increase in the untreated group (34). On the other hand, we have twice observed a significant transient increase in viral load approximately one month after de-worming, in particular, after praziquantel treatment in HIV–*Schistosoma* co-infection; this could be related to Th2 responses to dying schistosomes and to loss of the anti-inflammatory effects of schistosome-induced IL-10 (53,55). This may be important with regard to the treatment of helminths in HIV-infected women during pregnancy, as a transient rise in viral load might have the reverse of the desired effect on vertical HIV transmission. Modjarrad and colleagues compared changes in CD4 counts and viral load over an approximately 9-week period between participants who were treated for helminths (predominantly hookworm and *Ascaris*) and participants without helminths, who were untreated. They found little change in CD4 count or viral load in either group (51). In other studies, comparisons have been made, within helminth-infected groups, between those treated successfully and those with persistent infection. Wolday and colleagues (*Ascaris* and *Trichuris*) observed a decrease in viral load following successful treatment, and an increase with persistent infection (54), but no such effect was observed for the common species (*S. mansoni*, hookworm, *Strongyloides* and *Mansonella*) in our study in Uganda. In fact, in Uganda, particular interest again attached to *Mansonella.* This species was not susceptible to the treatments given, and, being regarded as non-pathogenic, was not specifically treated. Persistence of infection was associated with a decline in viral load, and spontaneous clearing of infection with an increase (52).

**Table 3:** Associations between helminths and worm infections: prospective studies of treatment

Number of participants and helminth species	Treatment	Follow-up time	Comparison made	Change in CD4 with treatment	Change in viral load (VL) with treatment	Country (ref)	Comments
130 participants with *S. mansoni* and/or *S. haematobium*	Prazi 40 mg/kg	3 months	Untreated vs. treated	No significant change in either group	No change in treated group; increase in untreated group	Zimbabwe (34)	*P* = 0·03 for difference in change in viral load between treated and untreated groups
54 participants (predominantly*Ascaris* and hookworm)	Two treatments, 1 mth apart, with alb (3 days,400, 200, 200 mg) and prazi (40 mg/kg, divided)	9 weeks	Uninfected vs. infected	No significant change in either group	No significant change in either group	Zambia (51)	VL declined after treatment in 6 people with high intensity infection
234 participants with helminths	Alb 400 mg stat^a^	6 months	Successfully treated vs. persistently infected	NS	NS	Uganda (52)	^a^Plus additional specific treatment for *S. mansoni* or *Strongyloides* if indicated. In the cohort as a whole, CD4 count declined over 6 months (*P <* 0·001); viral load showed no significant change
97 Hookworm	Alb 400 mg stat			NS	NS		
159 *S. mansoni*	Prazi 40 mg/kg			Larger decrease if cleared (*P =* 0·05)	NS		
53 *Strongyloides*	Alb 400 mg bd 3/7			NS	NS		
40 *Mansonella*	None			NS	Increase if cleared, decrease if persisted (*P =* 0·005)		
31 participants with helminths (predominantly *Trichuris* and *Ascaris*)	Three treatments, 3 mth apart, with alb 200 mg daily, 3 days; plus prazi (40 mg/kg, divided, for schistosomiasis)	6 months	Uninfected vs. successfully treated vs. persistently infected	No change in any group	Decrease in successfully treated group; increase in uninfected and persistently infected groups (*P =* 0·04)^b^	Ethiopia (54)	^b^*P*-value for comparison with successfully treated group.
30 men with *S. mansoni*	Single dose of prazi 40 mg/kg	1–15 months	None	Not reported	Increased	Kenya (56)	No correlation between changein VL and change in egg count or CCA concentration

*S. mansoni: Schistosoma mansoni; S. haematobium: Schistosoma haematobium*. Alb: albendazole. prazi: praziquantel. NS: no statistically significant difference between the groups compared.

Thus cross-sectional, retrospective and prospective data to date fail to support the hypothesis that helminth infection, in general, promotes HIV progression, but the findings reported by Kallestrup and colleagues, together with studies in a macaque model, point to a detrimental effect of schistosomiasis (12,34). In the macaque, effects were observed principally in association with initial viral inoculation and with the development of adult worms and egg-laying following new or renewed schistosome infection; this is perhaps in keeping with the tendency of schistosome eggs to induce pathological inflammatory responses. Further, there is some evidence that schistosomiasis may be associated with increased susceptibility to tuberculosis in people with HIV infection (57): if this is the case, schistosomiasis could promote HIV progression indirectly, by increasing susceptibility to additional co-infections with a more potent influence on viral replication (58,59). Schistosomiasis aside, there are some hints that high intensity infections with certain helminth species may be associated with higher viral loads, which may decline with treatment, but, to be convincing, these findings would need to be confirmed in larger studies. The vast majority of HIV-infected adults with helminths have low intensity infections, so the effects, if any, of low-intensity infections are pertinent. As suggested by Modjarrad and colleagues, the effects of high intensity helminth infections may be best explored, and most relevant, among children with HIV, where both worm burden and viral load tend to be high (51). There are hints, too, of differences in effect between helminth species, and even of possible benefits of helminth infection; these need to be investigated in more focused, prospective studies; uncertainty regarding the advantages and disadvantages of treatment suggest that placebo-controlled trials would be justified.

#### Effects of helminths on vaccination against HIV

No vaccine against HIV is yet ready for implementation (60), but there is evidence that helminth infection can alter both cellular and antibody responses to other, existing vaccines (61–69); suppression of responses and switching to a Th2 profile have both been observed. Effects may differ for vaccines given orally (where effects of intestinal helminths on the mucosa may suppress the development of the response (66,67)), compared with parenteral vaccines; and for live vectors (viral vectors, *Salmonella* or Bacille Calmette Guérin (BCG)), where there could be effects on the replication of the vector and hence the dose of vaccine antigen experienced (69). Results of on-going studies of interactions between helminths and existing vaccines, including the role and timing of de-worming in determining vaccine efficacy, are likely to be pertinent as HIV vaccines are developed, tested and implemented; however, it is unlikely that helminth infection accounts for the poor immunogenicity of vaccine candidates tested in Africa to date: recent phase 1 trials of DNA/modified vaccinia Ankara (MVA) vaccines showed poor immunogenicity in both Africa and Europe (70–72).

### Effects of HIV on helminths

HIV-induced CD4^+^ T-lymphocyte depletion, immune activation and changes in Th1/Th2 and regulatory responses should, in theory, affect the epidemiology of helminth-associated disease in co-infected individuals. Anticipated effects might include changes in immunopathology, reduced efficacy of treatment and impaired resistance to infection or re-infection. However, to date, there is surprisingly little evidence of effects of HIV on these parameters. Most information available is from *Schistosoma*, *Onchocerca* and *Strongyloides* infections, as follows.

Animal models of immunosuppression suggest that granuloma formation and consequently schistosome egg excretion might be reduced in HIV infection (73). Initial studies in humans supported this hypothesis (47,74,75) with evidence of reduced egg excretion in HIV-infected subjects. Recent case reports describe symptomatic schistosomal infection as an immune reconstitution phenomenon following antiretroviral therapy: these also imply that pathological responses to schistosomes and schistosome eggs are suppressed in advanced HIV disease and recover with reconstitution of the immune response (76,77). However, other studies have failed to demonstrate an effect of HIV on egg excretion or liver fibrosis (38,43,55,78). Similarly, efficacy of praziquantel, which is considered to act, in part, by exposing worms to immunologically mediated killing (79), has not been found to be impaired in HIV-infected people (75,80). Karanja and colleagues demonstrated a small effect of HIV on the acquisition of resistance to re-infection in heavily exposed Kenyan adults, which may in part be explained by suppression of post-treatment *S. mansoni*-specific cytokine and antibody responses (81–83).

Studies from Uganda have explored associations between *Onchocerca volvulus* infection and HIV status. Reduced *Onchocerca*-specific cytokine and antibody responses were found in HIV-co-infected individuals, which were more marked in subjects with lower CD4^+^ counts, but skin microfilarial densities were marginally lower in HIV-positive subjects and it is difficult to exclude confounding by differences in exposure, or reduced onchocercal fecundity in the presence of HIV (84–86). Kipp and colleagues found more severe onchocercal skin disease in co-infected patients, but this finding was based on only six HIV-infected subjects (87). There was no effect of HIV on ivermectin efficacy (85).

The effect of HIV on *Strongyloides* infection is of particular interest because of the severe manifestations of disseminated *S. stercoralis* infection seen following immunosuppression by corticosteroids, malignancy and HTLV-1 infection. Although well described in HIV-infected subjects without evidence of other mechanisms of immunosuppression, the syndrome is less common than might be expected in regions of high co-prevalence (88). Animal models suggest that host immunosuppression facilitates dissemination by promoting a switch towards direct development of *Strongyloides* larvae into infective L3 larvae, thereby by-passing the free-living adult stage and allowing auto-infection of the host (89). Following on from this work, we studied *Strongyloides* development among HIV-infected adults in Uganda (90). Charcoal stool cultures positive for *Strongyloides* were dissected and the ratio of infective L3 larvae to free-living adults was assessed. We expected that subjects with lower CD4^+^ counts would have a higher proportion of infective L3 larvae, in keeping with animal models. In fact, the opposite was found, with a positive correlation between direct development and CD4^+^ count. This may explain the low incidence of hyperinfection in HIV-infected adults, and attests to differences in the nature of the immune deficit between advanced HIV infection and other immunosuppressive states: corticosteroid-induced immunosuppression is associated with prominent expression of regulatory cytokines, such as IL-10 (91), and HTLV-1 infection with depletion of type 2 cytokines (92), but in HIV infection, regulatory T cells may be preferentially destroyed (31) and type 2 cytokine responses may be relatively preserved (93). There was no association between CD4^+^ count and total number of larvae per culture in the Ugandan study (MB, unpublished observations), which further argues against either an increase in *Strongyloides* fecundity or burden. Thus although the association between *Strongyloides* and HIV observed in some co-prevalence studies may be due to increased susceptibility to *Strongyloides*, this may be mediated by a mechanism other than auto-infection. Recent case reports of symptomatic strongyloidiasis presenting as an immune reconstitution syndrome after initiation of antiretroviral therapy (ART) suggest a protective effect of HIV-induced immunosuppression on some aspects of *Strongyloides* pathogenesis (94–96).

Thus results to date indicate that effects of HIV on the pathological consequences of, and susceptibility to, helminth infections may be minor compared with effects on viral, bacterial and fungal infections, where a major component of the increased susceptibility is loss of control of replication: these organisms, unlike most helminths, complete their life-cycle within the host. The hypothesis, suggested above, that progressive impairment of immune regulation in HIV disease can render the host environment less conducive to the maintenance of parasitic helminth infections has not yet been investigated.

## CONCLUSIONS

Early hypotheses on potential mechanisms for helminth–HIV interactions were inspired by the Th1/Th2 hypothesis. These must now be superseded by models that take account of regulatory mechanisms which are being shown to have a potent effect on helminth-induced responses and, it emerges, on HIV.

There is epidemiological evidence for a bi-directional interaction between helminths and HIV; the direction of effects is not easy to disentangle in observational studies and there is a pressing need for well-designed, randomised, controlled intervention trials. These must take into account the possibility of differences in effect between helminth species, stage and intensity of infection; between effects on the acquisition, and effects on the progression, of HIV infection; and perhaps indirect effects of helminths mediated by a possible increase in susceptibility to additional co-infections such as malaria (7,8) and tuberculosis (11,57–59,68).

Consider the following suggestion. Host–parasite relationships (exemplified by *Mansonella*, perhaps also hookworm) that are characterized by minimal pathology, implying balanced interactions between inflammatory and regulatory responses, might not promote viral replication; their effect might be neutral or even protective with regard to HIV progression; their removal might promote viral replication by reducing regulatory control of transcription. They might, however, be associated with less effective immune responses after initial inoculation of virus or vaccine. Conversely, their host–parasite balance might be vulnerable to selective loss of components of the immune response during HIV progression, leading to lower prevalence with advancing HIV disease. Host–parasite interactions such as schistosomiasis, where inflammatory responses have persisted through evolution, perhaps due to a selective advantage for parasite egg excretion, may be more detrimental with regard to HIV infection and treatment may be beneficial in most circumstances.

The past decade has brought to light the interest and complexity of helminth–HIV interactions. The next decade provides opportunities for definitive studies.
